# Fruit Fly Larval Survival in Picked and Unpicked Tomato Fruit of Differing Ripeness and Associated Gene Expression Patterns

**DOI:** 10.3390/insects13050451

**Published:** 2022-05-10

**Authors:** Shirin Roohigohar, Anthony R. Clarke, Francesca Strutt, Chloé A. van der Burg, Peter J. Prentis

**Affiliations:** 1 Faculty of Science, School of Biology and Environmental Science, Queensland University of Technology, Brisbane, QLD 4001, Australia; a.clarke@qut.edu.au (A.R.C.); fstrutt@phau.com.au (F.S.); c.vanderburg@qut.edu.au (C.A.v.d.B.); p.prentis@qut.edu.au (P.J.P.); 2 Centre for Agriculture and the Bioeconomy, Queensland University of Technology, Brisbane, QLD 4001, Australia

**Keywords:** fruit fly, frugivorous larvae, induced defence, detoxification genes, fruit picking status, Tephritidae

## Abstract

**Simple Summary:**

Tephritid fruit flies are major pests to a wide range of fruits and vegetables. Female flies lay their eggs into the fruit where the resultant larvae cause damage and yield loss. To replace pesticide-based controls with more sustainable management approaches, we need to develop new generation technologies. Enhancing fruit resistance is a promising alternative but it has received limited research attention. In this study, we examined larval survival and gene expression changes of *B. tryoni* larvae and tomato fruit while the fruits were in different picking statuses (unpicked vs. picked) and ripening stages (colour break vs. fully ripe). We assessed larval survival in two time points of 48 h and 120 h after inoculation. The fruit picking status and ripening stage had a significant effect on *B. tryoni* larval survival at 120 h. The gene expression patterns were not affected by picking status; however, insect detoxification genes and plant-induced defence genes were upregulated across the treatments. Overall, we anticipated the lack of conformity between larval survival and gene expression as a result of overlooked candidate genes or critical sampling time points.

**Abstract:**

The larvae of frugivorous tephritid fruit flies feed within fruit and are global pests of horticulture. With the reduced use of pesticides, alternative control methods are needed, of which fruit resistance is one. In the current study, we explicitly tested for phenotypic evidence of induced fruit defences by running concurrent larval survival experiments with fruit on or off the plant, assuming that defence induction would be stopped or reduced by fruit picking. This was accompanied by RT-qPCR analysis of fruit defence and insect detoxification gene expression. Our fruit treatments were picking status (unpicked vs. picked) and ripening stage (colour break vs. fully ripe), our fruit fly was the polyphagous *Bactrocera tryoni*, and larval survival was assessed through destructive fruit sampling at 48 and 120 h, respectively. The gene expression study targeted larval and fruit tissue samples collected at 48 h and 120 h from picked and unpicked colour-break fruit. At 120 h in colour-break fruit, larval survival was significantly higher in the picked versus unpicked fruit. The gene expression patterns in larval and plant tissue were not affected by picking status, but many putative plant defence and insect detoxification genes were upregulated across the treatments. The larval survival results strongly infer an induced defence mechanism in colour-break tomato fruit that is stronger/faster in unpicked fruits; however, the gene expression patterns failed to provide the same clear-cut treatment effect. The lack of conformity between these results could be related to expression changes in unsampled candidate genes, or due to critical changes in gene expression that occurred during the unsampled periods.

## 1. Introduction

Under optimality models of plant defence, plants are predicted to invest more resources to defend tissues/structures with higher genetic fitness benefits than those with less [1]. Fruit, which produces and protects the seed, should have a very high fitness value to a plant and so should be heavily protected from herbivores and pathogens [2,3,4]. However, plants face an evolutionary trade-off with respect to fleshy fruit, as such fruit when ripe is also designed to attract and reward vertebrate seed disperses and so there is selective pressure to increase their attractiveness to herbivores [5,6,7]. The evolutionary solution is that fruit is commonly toxic when immature to protect the developing seed, but changes to being non-toxic when ripe so as not to deter seed dispersers [8,9,10]. However, while this general pattern of fruit defence is well known, the mechanistic details of fruit defence against frugivores, especially arthropod frugivores, is significantly less known. To date, most studies on plant defence mechanisms against herbivorous insects have focused on vegetative tissue [11,12,13,14] or flowers [15,16], but there is very limited work on the defence of fruit, particularly with respect to inducible defences.

Fleshy fruits are recognised as having constitutive defences, which include the mechanical defences of pericarp toughness and thickness [17,18] and chemical defences, such as toxic secondary metabolites and oils in fruit flesh [6,19,20] and peel [21,22]. High concentrations of phenols, tannins, and flavonoids in immature apple fruit inhibit *Cydia pomonella* (Linnaeus) larval development [23], while essential oils in the flavedo layer of *Citrus* peel protects those fruits against several species of tephritid fruit fly [24,25,26]. Both chemical and mechanical defensive strategies are stronger in unripe fruit and gradually decrease during fruit ripening [27,28,29,30,31].

In contrast to constitutive defences, data on fruit inducible defences against frugivores are scarce, although there is a significant body of data from plant pathogen research [32,33,34,35,36,37,38,39,40,41,42]. Inoculating unripe chilli with *Alternaria alternata* (Fr.) and *Steirochaete capsici* (Syd.) increased the amount of phenolic compounds in fruit [43], while tomato fruits infested with *A. alternata* had an increased vanillic acid concentration in the epicarp [44]. Similarly, the inoculation of ripe and unripe tomato fruits with *Botrytis cinerea* (Pers) saw the induction of the biosynthesis pathway, transcription factors, such as non-ripening *(NOR)*, ripening inhibitor *(RIN)* and never-ripe *(NR)*, and ethylene-regulated defence genes [45,46]. The only evidence at the molecular level for the inducible defence of fruit against insects comes from green olive dupes infested by maggots of the olive fruit fly, *Bactrocera oleae* (Rossi) [47]. In this system, 196 genes involved in plant response to biotic stress (such as wounding and pathogen attack), or abiotic stress (such as temperature fluctuation, drought and high NaCl) were differentially expressed in infested drupes compared to control drupes, while 19 proteins were also differentially expressed in infested fruits.

Many tephritids (Diptera: Tephritidae), which include *B. oleae*, are specialist frugivores, with the females laying eggs into fruit where the maggots feed and grow [48,49]. The fruit feeding habit makes them internationally significant pests of horticulture [50,51,52], and with increasing insecticide resistance [53,54,55] and the regulatory loss of older pesticides [56], novel controls are required. Fruit resistance against tephritids is well known [57,58,59,60] and the manipulation of this resistance through biotechnology offers a novel control approach [61]. However, the mechanisms of fruit resistance against fruit flies are generally unknown, except the hardening around the oviposition wounds (callus) in avocado [57,62,63], peel oils in *Citrus* [26,64], or a combination of pericarp toughness and tannin concentrations in cucurbits [17,65].

While there is evidence for fruit constitutive defence against fruit flies, the evidence for induced defences is significantly less. Older literature reports tephritid larvae having higher levels of mortality in unharvested fruit, compared with harvested fruits [66,67]. This suggests the presence of induced defences, which are disrupted by fruit picking, but the reported experiments were not explicitly testing this hypothesis. In the olive fly study of Corrado et al. (2012), putative defence gene families were identified, but again the question of induction was not explicitly tested, nor was a link made between gene expression and phenotype effect.

In this paper, we explicitly test the question of whether there is evidence for induced plant defences operating against fruit fly larvae, utilising both phenotypic and gene expression data. Extending on the novel work of Corrado et al. (2012), we run larval survival and then genotype experiments, so we can more accurately correlate differential larval survival with differential expression of both plant defence and insect detoxification genes. We use the Queensland fruit fly, *Bactrocera tryoni* (Froggatt), infesting tomato and *Solanum lycopersicum* (L.) H. Karsten as our model system. We utilise two tomato cultivars of known variation in their quality as larval hosts of *B. tryoni* [68], with the fruit at two ripening stages (colour break and fully ripe) and two harvest states (unpicked and picked). The ripening stage treatment was selected because the ripening category differentially affects *B. tryoni* larval survival in laboratory experiments [69]; while the harvest treatment was applied because of older literature reporting that tephritid maggots have higher levels of mortality in unharvested fruit compared with harvested fruits [65,66]. This suggests to us the presence of induced defences, which are disrupted by fruit picking and, if this is so, it should be detectable at both the phenotype and gene expression levels. The larval survival assessment of this paper records the impact of the different treatments on the survival of *B. tryoni* larvae at two time points in their development. The subsequent gene expression component of this work examines the relative expression of 28 target genes in larvae and 15 target genes in tomato for a subset of the treatments where phenotypic effects were most strongly expressed. The genes were selected based on a review of plant–herbivore molecular interaction studies [70] and, for the larvae, the selected genes are associated with the detoxification pathway, while for tomato, the selected genes contribute to receptors, signalling and defence pathways.

## 2. Materials and Methods

This paper combines the phenotypic and gene expression components by evaluating the effect of different fruit attributes (cultivar; harvest type; ripeness stage) on *B. tryoni* larval survival and then using samples from the larval survival experiment to test whether the observed phenotypic effects can be correlated with gene expression changes. For ease of flow, the Materials and Methods and Results sections treat the larval survival and gene expression studies as essentially independent, although they are directly linked, as the samples used in qPCR were those from the larval survival experiment.

### 2.1. Larval Survival

The phenotype study evaluated the effect of fruit from two tomato cultivars (Cherry and Roma), at two different ripening stages (colour break and fully ripe), unpicked or picked from the plant, on the survival of larval *B. tryoni*. The work was done by inoculating fruit with newly emerged neonate larvae and then assessing larval survivorship through destructive fruit sampling at two post-inoculation time points. The details of the different components of the study follow.

#### 2.1.1. Insect Source

In this study, we used laboratory reared flies (~4 generations from the wild) to obtain enough neonate larvae to run the experiment with adequate statistical power. Furthermore, laboratory reared flies were also used to avoid the confounding influence of diet variation in the study. *Bactrocera tryoni* were obtained as pupae from a colony maintained by the Queensland Government, Department of Agriculture and Fisheries, Brisbane. The adult flies were reared at 27 °C, 70% RH, and 12L: 12D and fed on protein hydrolysate, sugar, and water until sexually mature. After collecting the eggs using an egging device [71], the eggs were transferred by a brush to wet filter paper inside a Petri dish and incubated at 26 ± 1 °C, 70% RH, and 12L: 12D for 48 h to obtain the neonate larvae.

#### 2.1.2. Tomato Fruit

*Solanum lycopersicum* was chosen as the experimental host fruit, as it is known that both cultivars (Red Cherry and Roma) [68], and ripening stage (mature green, colour break, fully ripe) [69] influence *B. tryoni* offspring survival. Cherry and Roma cultivars were grown and maintained in a glasshouse (22–25 °C, 65% RH, natural light) at the Redlands and Queensland Crop Development Research Facility (27°31′29″ S, 153°15′02″ E), Cleveland, Southeast Queensland. No pesticides or fertilizers were applied to the tomatoes beyond the pre-mixed nutrients within the potting mix. The following two ripening stages, graded based on colour, were used in trials: fruit that had a mix of green and yellowish colour (breaker and turning results in colour break); fruit that was light red to red in colour (fully ripe) [72]. These visual colour categories and fruit ripening stages were found to be significantly different based on pericarp toughness and Brix in a prior study [69].

#### 2.1.3. Determination of Experimental Time Points

Infested fruits were destructively sampled at time points that corresponded with the presence of the 1st and 3rd instar larvae within the fruit. While the individuals within a larval cohort are not completely uniform in their development [73], based on our previous work, time points at 48 h and 120 h were determined as the best for sampling cohorts predominantly at their first/1st and last/3rd instars [68].

#### 2.1.4. Larval Inoculation and Survival Assessment

While the fruit was still on the plant, 40 neonate larvae (<1 h after egg hatching) were inoculated into each of the 40 fruits of each of the two ripening stages of each of the two cultivars. Inoculation was performed by making 2 mm-deep incisions in two sides of the fruit using a sterile surgical blade with 20 larvae, then gently transferred into each incision, which were subsequently covered by Elastoplast. Half of the inoculated fruits were picked immediately after inoculation and kept in the same condition as the unpicked fruits in a semi-controlled environment glasshouse (22 °C to 25 °C and 65% RH under natural light). Larval counts were performed at 48 and 120 h by destructively sampling 10 fruits for each treatment under the stereomicroscope and recording the number of surviving larvae. All the surviving larvae and samples of infested tomato tissue were collected, snap-frozen using liquid nitrogen and stored at −80 °C for the subsequent gene expression study.

We recognise that the artificial inoculation of fruit with neonate larvae is likely to immediately trigger plant defence responses, but as all the experiments needed to start with a known and consistent number of larvae, this could not be avoided. However, as the inoculation method was identical across all the treatments, we believe that any significant treatment effect subsequently detected, at either the phenotype or genotype level, can be attributed to the treatment rather than the initial inoculation process.

#### 2.1.5. Data Analysis

Data analyses were performed using R statistical software (version 3.5.1 2018-07-02) and graphs were generated using R or Sigma Plot version 14. To examine if the treatments significantly affected *B. tryoni* larval survival, a three-way analysis of variance (ANOVA) was performed for each of the two sampling times (independent treatments: cultivar, ripeness stage, fruit picking status; dependent data: number of surviving larvae at the sampling time). Cultivar was found to have no significant effect on larval survival (supporting Table S1) and was, thus, excluded from the independent factors. Subsequently, separate two-way ANOVAs for each of the two sampling times were performed with the independent treatments of ripening stage and fruit picking status. The interaction effects are presented in the results where significant. In the cases where higher level interactions are significant (which happened once in our data), the lower-level effects should be interpreted cautiously [74], but it is not statistically inappropriate to include them [75]. Levene’s test of homogeneity of variance was performed prior to ANOVA and data transformed if required.

### 2.2. Gene Expression

**Figure 1:**
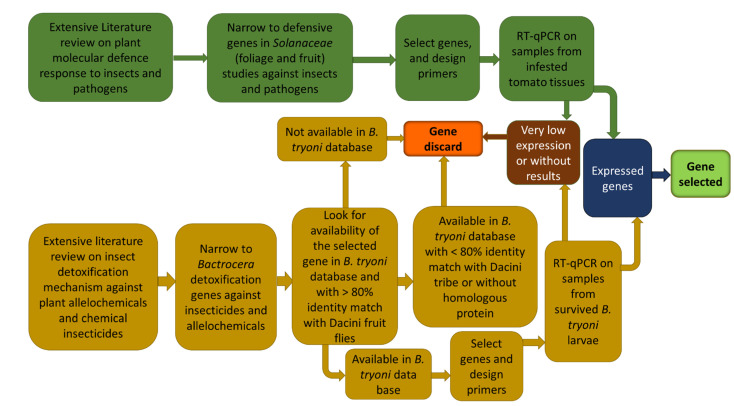
Schematic representation of the workflow used in the present study to choose inducible defence-related genes in tomato fruit and detoxification-related genes in *Bactrocera tryoni* (from Roohigohar et al., 2021).

The phenotypic trial found significantly higher larval survival from picked fruits compared to unpicked fruits in the colour-break stage of both tomato cultivars 120 h after inoculation (see Results). This outcome suggests fruit-induced defences that were disrupted by picking. To further address this question, a gene expression study was conducted on tissue from surviving larvae and infested tomato at the two-post inoculation sampling time points for the colour-break Roma treatment (Figure 1). The second tomato cultivar was not used because no cultivar effects were detected in the larval survival trial. The comparison of the expression level of 15 selected putative induced defence genes in tomato and 28 detoxification genes in *B. tryoni* were analysed from the following two perspectives: (i) comparison of gene expression in fruit and larval tissue from unpicked and picked tomatoes at the same time point; and (ii) comparison of gene expression in fruit and larval tissue from unpicked and picked tomatoes across the 48 h and 120 h time points. The details of the genes studied and RT-qPCR process follow.

#### 2.2.1. Tissue Collection

*Bactrocera tryoni* larvae (whole body) and infested tomato tissues were collected during the larval survival experiment by dissecting inoculated tomato fruits under the stereomicroscope at the QUT Genomics laboratory. Surviving larvae and tomato tissue of each individual fruit were collected into separate microtubes and immediately snap-frozen using liquid nitrogen and stored at −80 ℃. The procedures for RNA extraction, purification and cDNA synthesis are detailed in Roohigohar et al. (2021).

#### 2.2.2. Nominated Genes and Primers

The selection pipeline for identifying the target *B. tryoni* and *S. lycopersicum* genes for qPCR analysis is documented in Roohigohar et al. (2021) and summarized in Figure 1. Using this pipeline, 28 putative detoxification genes for *B. tryoni* and 15 putative induced defence genes for tomato were selected (Table 1). The procedures for primer design and primer checking for those genes are similarly detailed in Roohigohar et al. (2021). The primers used are provided in Table 2.

#### 2.2.3. qPCR Conditions

The qPCR reactions were performed in a LightCycler^®^96 Instrument (Roche) using a SensiFAST SYBR No-ROX Kit (BIO-98020). Each reaction contained 10 µL of SensiFAST SYBR, 0.8 µL each of forward and reverse primers (10µM), 0.5 µL of cDNA and 7.9 µL of H_2_O, with the final volume of 20 µL. As the negative controls, we used a no template control (NTC) and no-primer control reactions with two technical replications. The reactions were run with the following cycles: 1 cycle for polymerase activation at 95 °C for 2 min then 40 cycles at 95 °C for 5 s for denaturation, then 60–65 °C for 15–30 s for annealing/extension.

#### 2.2.4. Data Analysis

To analyse the qPCR data, *RS10B*, *RL18A*, *RT15* and *RT14* (encoding ribosomal proteins) from *B. tryoni* and *FPPS1* and *IDI1* (carotenoid biosynthesis function) from *S. lycopersicum* were used as housekeeping genes (internal control) by calculating their geometric mean, according to their stable cycle threshold during the experimental conditions. The relative expression level of the target genes was analysed using the 2^−ΔCT^ method [76]. The expression differences between the genes from different treatments were compared using an unpaired *t-*test (Welsh’s *t*-test for normal distribution) or a Mann–Whitney U test for non-normal data [77,78]. The Shapiro–Wilk normality test was performed prior to the final analysis to check the data distribution. The analysis was carried out in R statistical software (version 3.5.1 2018-07-02) and graphs were generated in Sigma Plot version 14.

## 3. Results

### 3.1. Larval Survival

**Figure 2:**
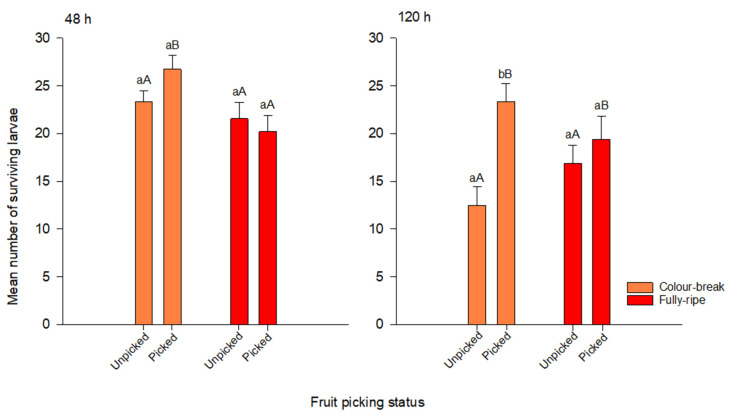
Mean (±1 SE) *Bactrocera tryoni* larval survival in tomato fruit at two ripening stages (colour break and fully ripe) and of two different picking states (unpicked or picked), at 48 h and 120 h after larval inoculation (starting *n* = 40 for each ripening × picking status treatment). Each time point includes the fruit picking state compared for each ripening stage (columns surmounted by the same lower-case letter are not significantly different at *p =* 0.05); fruit ripening stage compared within the picking state (columns surmounted by the same upper-case are not significantly different at *p =* 0.05).

After 48 h, when the majority of larvae were first instar, fruit ripening stage had a significant effect on larval survival, with mean larval survival in colour-break fruit significantly higher than in fully-ripe fruit (F_1, 79_ = 7.64, *p* = 0.007) (Figure 2). Picking status and the interaction effect were not significant (Table 3).

At 120 h, when most larvae were third instar, there was a significant interaction effect between the ripening stage and picking status on larval survival (F_1, 79_ = 4.15, *p* = 0.045). In the colour-break stage, larval survival was higher in picked fruits compared with unpicked fruits. However, picking status had no significant effect on larval survival in fully-ripe fruits (Figure 2). As a primary effect, picking status had a strong, significant effect on larval survival (F_1, 79_ = 10.61, *p* = 0.002), with larval survival significantly higher in picked tomatoes when compared with unpicked tomatoes after 120 h. The ripening stage as a primary effect was not significant (Table 3).

### 3.2. Comparative Gene Expression

#### 3.2.1. Differential Gene Expression in Picked and Unpicked Roma Tomato Fruit

##### Within a Sampling Period across Picking States

*48 h, picked vs. unpicked*

**Figure 3:**
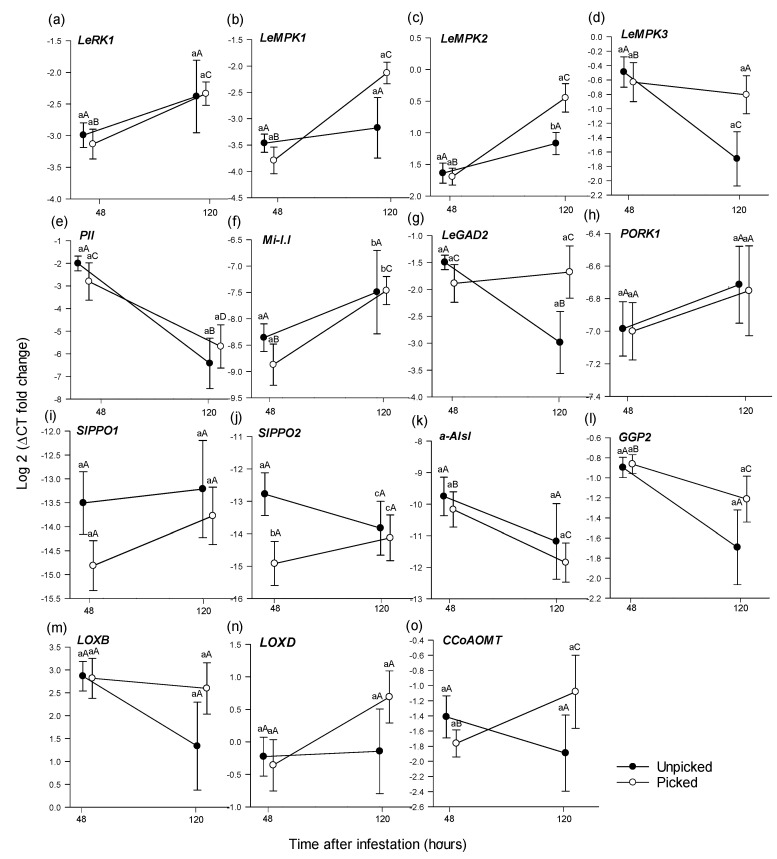
Mean (±1 SE) relative expression (log 2 of 2^−ΔCT^) of 15 putative induced defence genes (**a**–**o**), as assessed using RT-qPCR, from Roma tomato at the colour-break ripening stage infested by larvae of *Bactrocera tryoni*. Comparisons are of genes extracted from fruit at two time periods (48 h and 120 h) after larval inoculation and of two picking states (unpicked and picked); *n =* 10 fruit for each time point and status. Lower-case letters reflect significance or otherwise in the comparison across picking status within a time point; upper-case letters reflect significance or otherwise in the comparison within picking status across time points.

After 48 h, only *SIPPO2* was significantly differentially expressed between the two picking states, being higher in unpicked fruit than picked fruit (*t* (11) = −2.238, *p* = 0.047) (Figure 3j). Of the other 14 genes, none showed significantly different expression (Figure 3, Table 4).

*120 h, picked vs. unpicked*

One-hundred and twenty hours after larval inoculation, *LeMPK2* was expressed significantly higher in picked fruit compared with unpicked fruit (*z =* −2.419, *p* = 0.016) (Figure 3c). Again, the differences in expression of the other genes were not significant (Figure 3, Table 4).

##### Across Sampling Periods within a Picking State

*48 h vs. 120 h, unpicked*

In unpicked fruit, *PII* (*t* (18) *=* 2.457, *p* = 0.024)*, LeMPK3* (*t* (18) = 2.496, *p* = 0.025) and *LeGAD2* (*z =* −2.449, *p =* 0.017) were expressed significantly higher 48 h after inoculation compared with 120 h (Figure 3d,e,g); other genes did not exhibit significantly different expression (Figure 3, Table 4).

*48 h vs. 120 h, picked*

For picked fruit, *LecRK1*(*z =* −2.343, *p* = 0.021), *LeMPK1* (*t* (18) = −4.255, *p* < 0.001), *LeMPK2* (*t* (10) = −3.142, *p* = 0.010), *Mi-1.1* (*z* = −2.873, *p* = 0.004) and *CCoAOMT* (*t* (11) = −2.301, *p* = 0.030) were expressed significantly higher 120 h after infestation than at 48 h (Figure 3a–c,f,o), while *PII* (*z* = −2.419, *p* = 0.022) and *a-AIs1* (*z* = −2.041, *p* = 0.041) expressed significantly higher at 48 h than at 120 h (Figure 3e,k, Table 4).

#### 3.2.2. Differential Gene Expression in *B. tryoni* Larval Tissue

##### Within a Sampling Period across Picking States

*48 h, larvae from picked vs. unpicked fruit*

**Figure 4:**
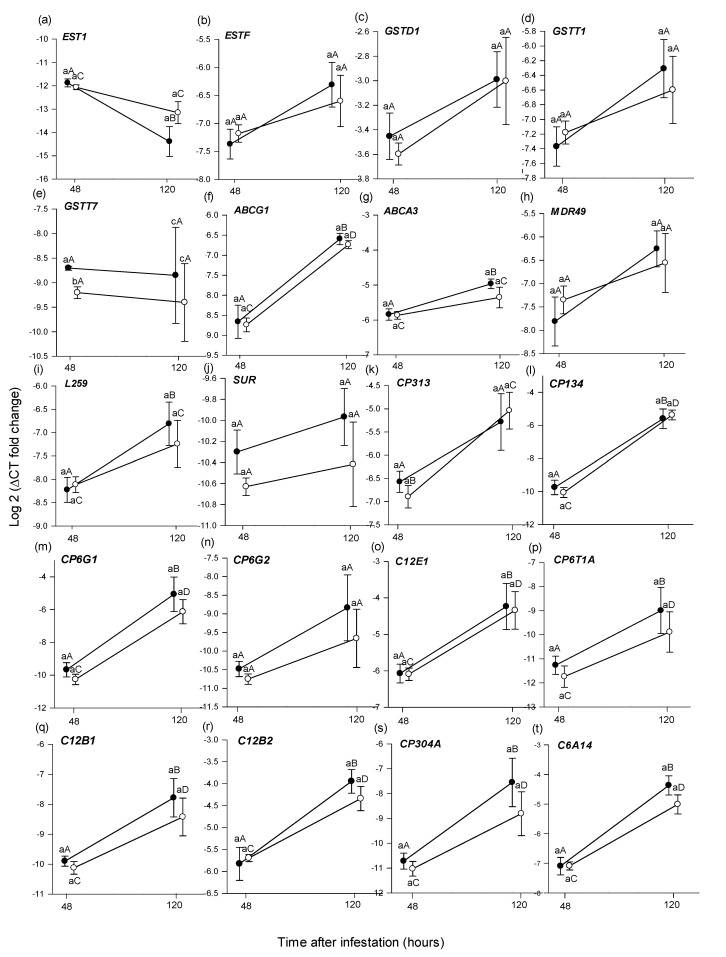

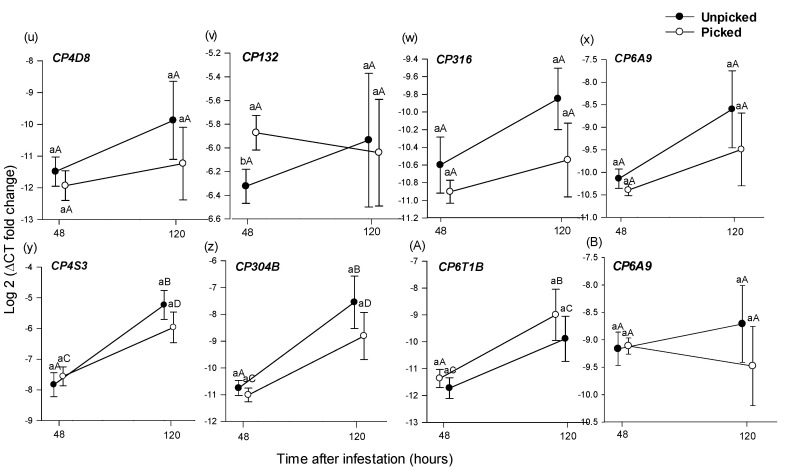
Mean (±1 SE) relative expression (log 2 of 2^−ΔCT^) of 28 putative detoxification genes (**a**–**z**,**A**,**B**), as assessed using RT-qPCR, from *Bactrocera tryoni* larvae extracted from Roma tomato at the colour-break ripening stage. Comparisons are of genes extracted from larvae sampled from fruit at two time periods (48 h and 120 h), after larval inoculation and of two picking states (unpicked and picked); *n = 8* fruit for 48 h and *n* = 7 for 120 h time points and picking status. Lower-case letters reflect significance or otherwise in the comparison across picking status within a time point; upper-case letters reflect significance or otherwise in the comparison within picking status across time points.

At 48 h, from the 28 selected *B. tryoni* genes, *GSTT7* was expressed significantly higher in larvae from unpicked versus picked fruit (*t* (14) = −4.002, *p* = 0.047) (Figure 4e) and CP*132* was expressed significantly higher in larvae from picked versus unpicked fruit (*t* (14) = 2.203, *p* = 0.040) (Figure 4v). No other genes exhibited significantly different expression (Figure 4, Table 5).

*120 h, larvae from picked vs. unpicked fruit*

There was no significant difference in the expression level of the 30 larval detoxification genes after 120 h in picked versus unpicked fruits (*p* > 0.05) (Figure 4, Table 5).

##### Across Sampling Periods within a Picking State

*48 h vs. 120 h, larvae from unpicked fruit*

The expression level of 15 genes from the larvae from unpicked fruits varied significantly between 48 and 120 h. *EST1* was expressed significantly higher at 48 h (*t* (10) = 5.654, *p* < 0.001) (Figure 4a), while *ABCG1* (*t* (12) = −7.336, *p* < 0.001)*, ABCA3* (*t* (12) = −3.976, *p* = 0.002)*, L259* (*z* = −2.619, *p* = 0.009)*, CP134* (*t* (6) = −2.668, *p* = 0.036)*, CP6G1* (z = −3.003, *p* = 0.003)*, C12E1* (*t* (6) = −2.491, *p* = 0.046)*, CP6T1A* (*z* = −2.108, *p* = 0.040)*, CP6T1B* (*z* = −2.236, *p* = 0.020)*, C12B1* (*z* = −2.747, *p* = 0.006)*, C12B2* (*t* (7) = −3.879, *p* = 0.005), *CP304A* (*z =* −3.002, *p* = 0.003)*, C6A14* (*t* (6) = −3.903, *p* = 0.007)*, CP4S3* (*z =* −2.*875, p* = 0.004) and *CP304B* (*z =* −2.364, *p* = 0.018) were all expressed significantly higher after 120 h (Figure 4f,g,i,l,m,o–t,y,z, A, Table 5).

*48 h vs. 120 h, larvae from picked fruit*

The expression level of 11 genes from the larvae from picked fruits varied significantly between 48 and 120 h. *ABCG1* (*t* (8) = −9.787, *p* < 0.001)*, CP313* (*t* (6) = −2.970, *p* = 0.02)*, CP134* (*t* (6) = −5.289, *p* = 0.001)*, CP6G1* (*z =* −3.130, *p* = 0.002)*, C12E1* (*t* (6) = −2.630, *p* = 0.030)*, C12B1* (*z =* −2.492, *p* = 0.013)*, C12B2* (*t* (6) = −3.612, *p* = 0.010), *CP304A* (*z =* −2.489, *p* = 0.015)*, C6A14* (*t* (6) = −2.942, *p* = 0.002)*, CP4S3* (*t* (6) = −2.432, *p* = 0.048) and *CP304B* (*z =* −2.236, *p* = 0.029) were all expressed significantly higher after 120 h (Figure 4f,k–m,o,q–t,y,z, Table 5).

## 4. Discussion

### 4.1. Results Summary

We evaluated the phenotypic effects of tomato fruit ripening stage and fruit picking status (unpicked vs. picked) on *B. tryoni* larval survival. Larval survival was influenced by the fruit ripening stage but not picking status at 48 h (better survival in colour-break fruit over fully-ripe fruit), and by an interaction of picking status and ripening stage at 120 h (better survival in picked colour-break fruit and picked and unpicked fully-ripe over unpicked colour-break fruit). The larval detoxification genes were upregulated at 120 h, with minimal difference if the fruit was on or off the plant. Similarly, there were only minimal differences in the expression patterns of the tomato defence genes between fruit on or off the plant, and where differences did occur, most were detected in picked fruit and so may have been associated with the picking process rather than larval infestation. The next sections of the discussion probe these results more fully.

### 4.2. B. tryoni Larval Survival in Tomato Fruit of Varying Ripeness and Harvest Status

The ripening stage and picking status both had a significant influence on larval survival at different time points. At 48 h, the larvae in colour-break tomatoes had greater survival than those in fully-ripe fruit. Several studies on frugivorous insects have found that fruit ripeness can have a strong impact on larval survival [60,79,80,81,82]. In an earlier, laboratory-based study, we reported similar larval survival results in Roma tomatoes and concluded that fully and over-ripe tomato fruit are not good quality nutritional hosts for *B. tryoni* larvae because the fruit starts breaking down before larval development is completed [69]. Fruit picking status had a significant impact on survival after 120 h, but this was largely driven by the change in larval survival in the unpicked colour-break tomato, which was approximately half of that in the picked colour-break fruit. In fully-ripe fruit at 120 h, larval survival was also poorer in unpicked versus picked fruit, but not significantly. Similar patterns of lower larval survival in the fruit remaining on the plant versus in picked fruit have been previously reported in the tephritids *B. tryoni* [66], and *Rhagoletis pomonella* (Walsh) [67], and the moth *Carposina sasakii* (Matsumura) [83]. This pattern of larval survival is strongly suggestive of a slow-acting induced defence response, which was broken when the fruit was removed from the plant, but which was exhibited more strongly in colour-break fruit than fully-ripe fruit. If so, this agrees with many previous studies that show fruit defences are “turned off” when fruit is fully ripe [84,85]. Although this is the most parsimonious explanation, we cannot rule out other factors explaining the larval survival results. Notably, this might include changes in tomato primary metabolites (e.g., sugar, carbohydrates, organic acids), differentially changing during ripening in picked and unpicked fruit.

### 4.3. Larval Gene Expression Indicates a Detoxification Response in Both Picked and Unpicked Fruit

The increase in expression of larval detoxification genes with time observed in our study indicates that the larvae were exposed to plant defence chemicals that required detoxification. Frugivorous insects protect themselves against plant toxins using a three-phase detoxification system [86,87,88,89]. The phase I enzymes metabolize toxins; phase II enzymes help detoxify or modify the toxic by-products generated in phase I; while phase III proteins are involved in the active removal of conjugated toxins from the cell [90]. Most of the upregulated larval genes from both the picked and unpicked fruits were from phase I, indicating a strong enzymatic detoxification response is occurring with increasing time spent in the fruit [91]. One gene from the larvae in the picked fruit, and three genes from the larvae in the unpicked fruit, were phase III detoxification proteins. Phase III genes mostly encode ATP-binding cassette transporters (ABC transporters), which are involved in the excretion of toxins from the cell [88]. The upregulation of Phase I and Phase III genes have been observed in both frugivorous and herbivorous insects, in response to the toxic compounds found in their host plants [86,92,93,94].

The slightly higher number of differentially expressed Phase III genes in the larvae from the unpicked fruit may be evidence that these larvae were exposed to more or different tomato toxic compounds than larvae in picked fruit, but this assessment is highly inferential. In addition, a noticeably higher expression variation was observed in 23 out of 28 of larvae detoxification genes at 120 h, compared with 48 h (Figure 4). This interesting result may be related to the level of secondary compounds in fruits or the changes in the composition of other toxic compounds associated with fruit ripening, but further research is required to better understand this pattern. The overall larval gene expression patterns indicated that the detoxification response was similar in larvae from both picked and unpicked fruit. This result was surprising, as we expected higher expression of the detoxification genes in the larvae from the unpicked fruit and we observed lower survival of larvae in this treatment at 120 h post infestation. Picked and unpicked tomato fruits contain a variety of toxic secondary compounds [95], at least one of which is known to slow the development of fruit fly larvae [96], and these compounds may have elicited the expression of detoxification genes, independent of the picking treatment. If the larval detoxification response was triggered by toxins already present in the fruit before harvest, then it does not support the presence of an induced defence component in the plant’s anti-frugivore response. Alternatively, if there is an induced component to the tomato fruit defence, as suggested by the 120 h larval survival data for colour-break fruit, then the larval response to constitutive defences is masking this in the gene expression data. Additionally, studies of herbivorous insects feeding on plants of differing toxicity have found that only a small subset of detoxification genes are differentially expressed in response to varying levels of toxic compounds [94]. This infers the selection of candidate genes is critical for any such study. With only one prior arthropod frugivory study upon which to base our gene selection, it is possible we may have missed subtle gene expression changes in larvae between the picked and unpicked fruits because we did not choose the correct subset of candidate detoxification genes.

### 4.4. Tomato Gene Expression Patterns

At the same time point, the differential expression of the tomato defence genes was almost entirely unaffected by picking status; while within a picking treatment across time where differential gene expression did occur, it did so in an inconsistent fashion, which may have been as much related to the physical picking of the fruit as to the presence of fruit fly larvae. However, before beginning the tomato gene expression discussion, we need to re-address the fruit inoculation limitation. Fruit artificially infested with larvae might have immediately triggered the expression of tomato wound-induced resistance genes. This experimental inoculation procedure could not be avoided, as our all experiments had to start with a known number of neonate larvae. However, as the inoculation method was consistence across all treatments, and we believe that any significant inoculation effects on either phenotype or gene expression level can be attributed to the treatment rather than the initial inoculation process. The tomato gene expression data, therefore, support the larval gene expression findings that the detoxification response most likely occurs to the toxins already present in the fruit. These findings still do not, however, explain the higher larval mortality detected in colour-break, unpicked fruits at 120 h. If differential defence gene expression does occur in tomatoes following fruit fly larval infestation, and we did not detect it, then we believe there may be two possible reasons for this. As for the larval study, the first issue again may be our selection of candidate genes. In fact, a recent review concluded human bias can distort candidate gene choice in plants [97], and lead to the erroneous choice of candidate genes that are peripheral to the specific trait studied. All 15 candidate tomato genes were selected based on their known roles in plant defence, but prior studies have predominantly examined vegetative tissue and folivorous insects, or tomato fruit and pathogens [98,99,100,101]. Our omission of genes with critical roles in defence against frugivorous insects could have occurred if these genes do not overlap those involved in vegetative tissue or pathogen defence.

The second reason we may have failed to detect differential gene expression, if it was there, were the time points selected. Our time points of 48 and 120 h were chosen based on the optimal time for recovery of the different *B. tryoni* larval stages, but were not optimised for studying gene expression patterns. The 48 h sampling, particularly, may have been too late to observe major transcriptional changes in defence gene expression if they occurred. For example, olive drupes infested with *B. oleae* larvae had higher expression of two highly inducible defence genes (Oe-Chitinase I and Oe-PR27) within 24 h [47]; while inoculation of tomato fruit with conidia of *Colletotrichum gloeosporioides* (Penz.) triggered tomato transcript changes within 19 h [45]. Unfortunately, in our system, larvae are too small to recover from fruit with any level of accuracy prior to 48 h. Therefore, we suggest that future studies of this type separate the larval survival evaluation and gene expression components, rather than run them simultaneously as we did here, as the optimal timing to measure one effect (e.g., larval survival, measured over multiple days) may not be optimal for measuring another (e.g., gene expression, potentially measured within a day).

## 5. Conclusions

Taken together, our findings indicate that *B. tryoni* larvae were under greater stress in the unpicked tomato fruit than picked fruit, as reflected by the differential larval survival at 120 h. This supports our initial hypothesis that an induced plant defence was occurring. However, at the molecular level, we failed to detect a differential expression signal that any induced fruit defence was occurring. With the current state of molecular knowledge of fruit-induced defence pathways against frugivorous insects, we cannot separate the following two alternatives for explaining this disparity: firstly, that the molecular data are correct and differential larval mortality was due to fruit constitutive defences or (non-defensive) fruit metabolic changes; or secondly, the molecular data are incorrect because we have missed critical defence genes and/or critical gene expression time points. While experimentally frustrating, this study nevertheless lays the groundwork for further experimentation in this system, and so starts unravelling the “black box” that is fruit fly larval mortality within fruit [49]. This knowledge is foundational to any future attempts at fruit resistance breeding for sustainable pest management.

## Figures and Tables

**Table 1:** Genes selected for studying plant-induced defence/insect-detoxification interactions that occur between *Bactrocera tryoni* larvae feeding in *Solanum lycopersicum* fruit.

Gene Family/Pathway	Gene Symbol	Gene Function
*B. tryoni* detoxification pathway genes		
Cytochrome P450	CP6A9, CP313, CP134, CP4D8, CP6G1, C12E1, CP6T1A, CP6T1B, C12C1, C12B1, C12B2, CP304A, C304B, CP306, C6A14, C4AC2, CP4S3, CP132, CP316, CP6G2	Catalysis of oxidative reactions during endogenous and exogenous metabolism and metabolism of xenobiotics and plant allelochemicals
Carboxylesterase	EST F, EST 1	Hydrolysis drugs, environmental toxicants, and insecticides
Glutathione S-transferase	GST D1, GST T1, GST T7	Detoxification of endogenous and xenobiotic compounds
ATP-binding cassette (ABC) transporters	ABCG1, ABCA3, SUR, L259, MDR49	Facilitate cellular excretion of insecticides or metabolites
***S. lycopersicum*** **defensive pathway genes**		
Receptor-like kinase	PORK1, LecRK1	Phytophagous arthropod attacks perception in plant tissue
D-mannose/L-galactose	GGP2	Oxidative stress response in plant against abiotic and biotic stresses
Mitogen-activated protein kinase	LeMPK1, LeMPK2, LeMPK3	Plant signal transduction in response to biotic and abiotic stresses
Lipoxygenase	LOXB, LOXD	Plant defence response against pathogens and herbivores
Gamma-aminobutyric Acid	LeGAD2	Increases plant resistance to insect herbivory
Polyphenol oxidase	SlPPO1, SlPPO2	Plant defence response against pathogens and insects
Proteinase inhibitor	PII, a-AIs1	Inhibiting insects’ digestive enzymes
Caffeoyl-CoAO-methyltransferase	CCoAOMT	Plant phytoalexins against herbivores and pathogens
Resistance (R) gene	Mi-1.1	Plant resistance against pests

**Table 2:** Primer pairs for genes selected for studying the plant-induced defence/insect detoxification interactions occurring between *Bactrocera tryoni* larvae feeding in *Solanum lycopersicum* fruit. The primer development and primer check processes for these genes are provided in Roohigohar et al. (2021).

Gene Symbol	Forward Sequence 5′-3′	Reverse Sequence 5′-3′
*B. tryoni* detoxification pathways genes and primers		
*GST D1*	GCCGATTTCACCACGTATGC	GCGTGTATCGCTGAAACGTC
*GST T1*	TTAGCACCATAGACGTGGCG	TGG GCAATACTGCGGAACTT
*GST T7*	TGGCCGGTGATCAGTTGAAA	GCTGATCGACCATAGCACGA
*EST F*	AGCTAAACCTTCCACCACGG	CACCCATTGCAAAGCCAGAC
*EST 1*	CGCTGTTTACGCATTCCTCG	AGCGGACGCATACTCATAGC
*SUR*	TTGCTCAAGGCAAAGCGAAC	CATCGTCATCCGTCTGCTCA
*ABCG1*	TTCTTTGTCGGTGCTACGCT	ATGGGCGTTCCAAGCCATAA
*ABCA3*	GGGAATAGCGATTGCGGGTA	CGCTTCTTCCATGTGATGCG
*L259*	CAGGAGCCAGCACGTAAAGA	GGTCCAATGACGGCCACTAA
*MDR49*	TGAGGCAACCTCGGCTTTAG	CCGAGCGCATAAGTTCAACG
*CP6A9*	GTATCGCTTGCAACTCGCTG	CGCACGATGCGCATAAAGAA
*CP313*	AACACTTCAAACCGGAGGCA	CTCCAGCTGACACAACGGAT
*CP134*	AGGGCATTTCGATTGGCAGA	TCACCCGCATCGTTTCGTTA
*CP4D8*	ATTTACTCGCACGCCATCCA	CGGCACACTGGGATAGAGAC
*CP6G1*	TGGACGAAGTGTTGCGCTTA	GGATCGAAAGTGTCCGGGTT
*C12E1*	ATGTGGACTTGGAGAACGCA	TCCATTTCCCGAATGGCAGT
*CP6T1A*	TGCATAATCATGCGCTGCTG	GTCTCCAGCTTACCGCCAAT
*CP6T1B*	CGCGCACATCTTTACTCAGC	GCCAGTAACAAGAAAGCGGC
*C12B2*	CAGCTTTCGGATGTTGCGAG	ACCGGCCAGATGGTTTCATT
*C12B1*	TACGCACACTGCCGAAAGAT	TTCCGGACAAGCACTCTCAC
*CP306*	CCTGCTCGCGCTATTAGTCA	TTCAAGAATTCCCGCACCGA
*CP304A*	AGCGTCGTGCTGACGATTAT	GTATGCCCATTCGCGTGTTC
*C6A14*	ACACTGCGGAAATACACGGT	CGAAACGATCGGGTTCAGGA
*CP4S3*	AAGCGCTGAAGGTACTGCAT	AAGTGTCGACTTCTTCGCGT
*CP132*	AGCACACCTCTTCAATCCCG	CTGCGATCTCAGCATAACGC
*CP316*	AATCGGTTCGGTGCAGAAGT	ATGATCTGCGCTGTGTAGCA
*CP304B*	TGAGGTCGTAGGTAGAGGGC	GCTCCGTGTCTACCAATGCT
*CP6G2*	CGCGCTGTGTTCAAGTTCAG	CGCAGAAACTCGGTAGAGGT
***S. lycopersicum* defensive pathways genes and primers**		
*PORK1*	AGACCCTCAATGAAAGAGGTA	GGTGGAGCTAGAAGTGAGACA
*slPPO1*	GTGGACAGGATGTGGAACGA	CTTCTTGGTGTCCAGGCAGT
*slPPO2*	AGTTGTTGCCCTCCTGTACC	CCCTCATTCGACTCGTAGCC
*LecRK1*	CTTTGCAGGCATCGTGCTTT	GCGCAAAGGTGAAGGGATTG
*PII*	TGGTGTACCAACAAAGCTTGC	GCATTTGTACAACAAAGCCCA
*LeMPK1*	GATGGTTCCGTTCCGCAAAC	GAACCTGCCACCATGGCTTA
*LeMPK2*	GCGCTTGCTCATCCTTACCT	AATCCAACAGCAAACGAGCG
*LeMPK3*	CGCCCTTACGAAGGGAGTTT	ACTTTAGCCCACGGAGAAGC
*GGP2*	CCTCCACTTCCAGGCGTATT	GCATCAGACAAATCACGGGC
*Mi-1.1*	AAAGCTCACCAGTGGATCGG	CCATGCACGAAGGTCGAAAC
*LOXB*	GCGTTTAAGGCTTTGTGCGA	GTAGGCCTTGACCATCCGTT
*LOXD*	GCAGATCGCTAAAGCACACG	GCGCTTAACTGCCTATGTGC
*CCoAOMT*	ACCAAATGATTGACGACGGC	TCCGTTCCAAAGGGTGTTGT
*LeGAD2*	TGAGCCCTGAGAAAGCTGTG	GGAGTGTCCCACCCTGTTTC
*a-AIs1*	AAGTGCCTCACCAACACCAT	CAGAATTCGTCGCGGATGGA

**Table 3:** Two-way analysis of variance results for *Bactrocera tryoni* larval survival in tomato fruit of two ripening stages (colour break and fully ripe) and two picking states (picked or unpicked). Separate ANOVAs are presented for the fruits that were destructively sampled 48 and 120 h after larval inoculation.

Treatment/Interaction	df	F	*p*
**48 h after inoculation**			
Ripening stage	**1, 79**	**3.528**	**0.019**
Picking status	1, 79	0.461	0.499
Ripening * picking status	1, 79	2.475	0.120
**120 h after inoculation**			
Ripening stage	1, 79	0.012	0.913
Picking status	**1, 79**	**10.61**	**0.002**
Ripening * picking status	**1, 79**	**4.154**	**0.045**

**Table 4:** The statistical analysis results of differential gene expression in unpicked and picked colour-break Roma tomato fruit inoculated with *Bactrocera tryoni* larvae and dissected at 48 h and 120 h after inoculation. The comparative analyses were performed between the following: (A) gene expression from infested tomato tissue sampled from unpicked versus picked tomato fruit 48 h after larval inoculation; (B) gene expression from infested tomato tissue sampled from unpicked versus picked tomato fruit 120 h after larval inoculation; (C) gene expression from infested tomato tissue sampled from unpicked tomato fruit 48 h versus 120 h after larval inoculation; and (D) gene expression from infested tomato tissue sampled from picked tomato fruit 48 h versus 120 h after larval inoculation. Analyses were unpaired *t*-test or Mann–Whitney U test, depending on the error distribution of the data. The 15 selected genes are putatively associated with plant-induced defence response and are as follows: (1) plant perception, such as receptor-like kinase pathway (*PORK1* and *LecRK1*); (2) signalling transduction, such as D-mannose/L-galactose pathway (*GGP2*), mitogen-activated protein kinase pathway (*LeMPK1, LeMPK2 and LeMPK3*), lipoxygenase (*LOXB* and *LOXD*); (3) GABA signalling pathway (*LeGAD2*); (4) genes with anti-nutritional activity (*SlPPO1-2, PII* and *a-AIs1*); (5) CoA O-methyltransferase (*CCoAOMT*); (6) tomato resistance *R* gene (*Mi-1.1*). *: *p* < 0.05.

A-Tomato Fruit 48 h	Unpicked	Picked				
Gene symbol	Mean of 2^−ΔCT^ (*n* = 10)		*t-value/z-value*	*df*	*p-Value*	Expressed higher
*PORK1*	0.0084	0.0083	−0.006	18	0.994	
*slPPO1*	1.86 × 10^−4^	5.77 × 10^−5^	−1.568	-	0.121	
***slPPO2***	**2.98 × 10^−4^**	**7.61 × 10^−5^**	**−2.238**	**11**	0.047 *	***Unpicked***
*LecRK1*	0.1370	0.1318	−0.245	-	0.586	
*PII*	0.3110	0.2877	−0.219	18	0.828	
*LeMPK1*	0.0968	0.0860	−1.423	-	0.212	
*LeMPK2*	0.3408	0.3228	−0.327	18	0.623	
*LeMPK3*	0.7928	0.7578	−0.173	18	0.864	
*GGP2*	0.5486	0.5602	0.222	18	0.826	
*Mi-1.1*	0.0036	0.0035	−1.937	-	0.064	
*LOXB*	9.0365	11.2538	0.497	13	0.627	
*LOXD*	1.0514	1.1575	−0.320	-	0.628	
*CCoAOMT*	0.4390	0.3162	−1.337	18	0.197	
*LeGAD2*	0.3698	0.3422	−0.305	12	0.765	
*a-AIs1*	0.0025	0.0015	−0.489	-	0.840	
**B-Tomato tissue** **120 h**	**Unpicked**	**Picked**				
*PORK1*	0.0109	0.0108	−0.024	18	0.980	
*slPPO1*	0.0006	0.0001	−0.916	-	0.361	
*slPPO2*	0.0003	0.0001	−0.239	-	0.708	
*LecRK1*	0.2125	0.7589	−0.874	9	0.404	
*PII*	0.0901	0.26787	0.697	10	0.501	
*LeMPK1*	0.1596	0.2477	2.083	18	0.051	
***LeMPK2***	**0.4760**	**0.8220**	**−2.419**	**-**	0.016 *	***Picked***
*LeMPK3*	0.6701	0.6701	1.754	18	0.096	
*GGP2*	0.4207	0.4837	0.454	18	0.654	
*Mi-1.1*	0.0229	0.0066	−0.677	-	0.619	
*LOXB*	5.9229	11.8689	−0.939	-	0.421	
*LOXD*	3.5808	2.1924	−0.484	10	0.639	
*CCoAOMT*	0.4748	0.6363	0.652	18	0.522	
*LeGAD2*	0.2815	0.4583	−1.637	-	0.121	
*a-AIs1*	0.0275	0.0004	−0.209	-	0.850	
**C-Tomato tissue** **Unpicked**	**48 h**	**120 h**				
*PORK1*	0.0084	0.0109	−1.008	13	0.332	
*slPPO1*	1.86 × 10^−4^	0.0006	−1.408	-	0.189	
*slPPO2*	2.98 × 10^−4^	0.0003	−0.213	-	0.834	
*LecRK1*	0.1370	0.2125	−0.995	-	0.345	
***PII***	**0.3110**	**0.0901**	**2.457**	**18**	0.024 *	***48 h***
*LeMPK1*	0.0968	0.1596	−1.986	12	0.069	
*LeMPK2*	0.3408	0.4760	−1.870	18	0.077	
***LeMPK3***	**0.7928**	**0.6701**	**2.496**	**18**	0.022 *	***48 h***
*GGP2*	0.5486	0.4207	1.069	11	0.308	
*Mi-1.1*	0.0036	0.0229	−1.278	-	0.307	
*LOXB*	9.0365	5.9229	1.192	18	0.248	
*LOXD*	1.0514	3.5808	−0.894	9	0.394	
*CCoAOMT*	0.4390	0.4748	−1.159	-	0.241	
***LeGAD2***	**0.3698**	**0.2815**	**−2.449**	**-**	0.017 *	***48 h***
*a-AIs1*	0.0025	0.0275	−1.521	-	0.104	
**D-Tomato tissue** **Picked**	**48 h**	**120 h**				
*PORK1*	0.0083	0.0108	−1.049	18	0.308	
*slPPO1*	5.77 × 10^−5^	0.0001	−1.125	-	0.289	
*slPPO2*	7.61 × 10^−5^	0.0001	−0.821	-	0.596	
***LecRK1***	**0.1318**	**0.7589**	**−2.343**	**-**	0.021 *	***120 h***
***PII***	**0.2877**	**0.26787**	**−2.419**	**-**	0.022 *	***48 h***
***LeMPK1***	**0.0860**	**0.2477**	**−4.255**	**18**	0.0004 *	***120 h***
***LeMPK2***	**0.3228**	**0.8220**	**−3.142**	**10**	0.010 *	***120 h***
*LeMPK3*	0.7578	0.6701	0.441	18	0.664	
*GGP2*	0.5602	0.4837	0.877	13	0.396	
***Mi-1.1***	**0.0035**	**0.0066**	**−2.873**	**-**	0.004 *	***120 h***
*LOXB*	11.2538	11.8689	−0.090	18	0.928	
*LOXD*	1.1575	2.1924	−1.542	18	0.140	
***CCoAOMT***	**0.3162**	**0.6363**	**−2.301**	**11**	0.042 *	***120 h***
*LeGAD2*	0.3422	0.4583	−0.804	18	0.431	
***a-AIs1***	**0.0015**	**0.0004**	**−2.041**	**-**	0.041 *	***48 h***

**Table 5:** Statistical analysis results of differential gene expression in *Bactrocera tryoni* larvae reared in unpicked or picked colour-break Roma tomato fruit and collected in 48 h and 120 h after inoculation (when larvae were approximately in their 1st and 2nd instars). The comparative analyses were performed between the following: (A) gene expression from tissue of larvae sampled from unpicked versus picked tomato fruit 48 h after larval inoculation; (B) gene expression from tissue of larvae sampled from unpicked versus picked tomato fruit 120 h after larval inoculation; (C) gene expression from tissue of larvae sampled from unpicked tomato fruit 48 h versus 120 h after larval inoculation; and (D) gene expression from tissue of larvae sampled from picked tomato fruit 48 h versus 120 h after larval inoculation. Analyses were unpaired *t*-test or Mann–Whitney U test, depending on the error distribution of the data. The 28 selected genes are associated with insect detoxification pathways and are as follows: (1) cytochrome P450 (*CP6A9, CP313, CP134, CP4D8, CP6G1, C12E1, CP6T1A, CP6T1B, C12C1, C12B1, C12B2, CP304A, C304B, CP306, C6A14, C4AC2, CP4S3, CP132, CP316* and *CP6G2*); (2) carboxylesterase (*EST F* and *EST 1*); (3) glutathione S-transferase (*GST D1*, *GST T1* and *GST T7*); (4) ATP-binding cassette (ABC) transporters (*ABCG1*, *ABCA3*, *SUR*, *L259* and *MDR49*). *: *p* < 0.05.

A-Larvae Tissue 48 h	Unpicked	Picked				
Gene symbol	Mean of 2^−ΔCT^ (*n* = 8)		*t-value/z-value*	*df*	*p-Value*	Expressed higher
*GST D1*	0.1000	0.0820	−1.442	14	0.171	
*GST T1*	0.4758	0.3974	−1.195	14	0.251	
***GST T7***	**0.0023**	**0.0017**	**−4.002**	**14**	0.001 *	***Unpicked***
*EST F*	0.0071	0.0069	−0.135	14	0.894	
*EST 1*	0.00027	0.00023	−1.129	14	0.277	
*SUR*	0.0008	0.0006	−2.078	9	0.066	
*ABCG1*	0.0031	0.0024	−1.308	14	0.211	
*ABCA3*	0.0177	0.0174	−0.155	14	0.878	
*L259*	0.0036	0.0037	−0.184	-	0.864	
*MDR49*	0.0063	0.0067	0.116	-	0.955	
*CP6A9*	0.0009	0.0007	−1.216	10	0.252	
*CP313*	0.0119	0.0093	−1.096	14	0.291	
*CP134*	0.0014	0.0010	−0.231	-	0.867	
*CP4D8*	0.0004	0.0003	−0.547	14	0.592	
*CP6G1*	0.00160	0.00162	−0.347	-	0.779	
*C12E1*	0.0155	0.0151	−0.157	14	0.877	
*CP6T1A*	0.0003	0.0002	−1.042	14	0.314	
*CP6T1B*	0.0004	0.0003	−0.615	14	0.548	
*C12B2*	0.00046	0.00037	−0.615	12	0.549	
*C12B1*	0.0005	0.0004	−0.616	14	0.548	
*CP306*	0.00194	0.00193	−0.030	14	0.975	
*CP304A*	0.00035	0.000350	−0.022	14	0.982	
*C6A14*	0.0079	0.0073	−0.694	-	0.536	
*CP4S3*	0.0051	0.0058	0.459	14	0.652	
***CP132***	**0.0127**	**0.0169**	**2.203**	**14**	0.044 *	***Picked***
*CP316*	0.0007	0.0005	−0.243	8	0.246	
*CP304B*	0.00066	0.00053	−0.783	14	0.446	
*CP6G2*	0.00072	0.00059	−1.288	14	0.218	
**B-Larvae tissue** **120 h (*n* = 7)**	**Unpicked**	**Picked**				
*GST D1*	0.1353	0.1594	−0.694	-	0.536	
*GST T1*	0.7766	1.0905	−0.958	-	0.338	
*GST T7*	0.0108	0.0051	−0.447	-	0.710	
*EST F*	0.0162	0.0138	−0.343	12	0.737	
*EST 1*	7.13 × 10^−5^	1.48 × 10^−4^	1.494	12	0.160	
*SUR*	0.0011	0.0008	−0.822	12	0.426	
*ABCG1*	0.0106	0.0095	−0.960	12	0.355	
*ABCA3*	0.0328	0.0269	−1.148	12	0.273	
*L259*	0.0137	0.0099	−0.469	12	0.647	
*MDR49*	0.0173	0.0173	0.005	12	0.995	
*CP6A9*	0.0102	0.0059	−0.443	12	0.665	
*CP313*	0.0391	0.0374	−0.115	12	0.909	
*CP134*	0.0308	0.0270	−0.314	12	0.758	
*CP4D8*	0.0078	0.0038	−0.534	12	0.602	
*CP6G1*	0.1161	0.0278	−1.224	-	0.264	
*C12E1*	0.0827	0.0687	−0.418	12	0.682	
*CP6T1A*	0.0114	0.0048	−0.594	-	0.563	
*CP6T1B*	0.0103	0.0047	−0.572	-	0.577	
*C12B2*	0.0103	0.0047	−0.572	12	0.576	
*C12B1*	0.01033	0.0046	−0.572	12	0.577	
*CP306*	0.0062	0.0039	−0.419	12	0.682	
*CP304A*	0.0100	0.0061	−1.214	-	0.259	
*C6A14*	0.5602	0.0365	−1.250	12	0.234	
*CP4S3*	0.0381	0.0216	−1.021	12	0.327	
*CP132*	0.0249	0.0213	−0.294	12	0.773	
*CP316*	0.0012	0.0008	−1.456	12	0.170	
*CP304B*	0.0172	0.0101	−0.831	-	0.456	
*CP6G2*	0.0084	0.0046	−0.490	12	0.632	
**C-Larvae tissue** **Unpicked**	**48 h**	**120 h**				
*GST D1*	0.0961	0.1353	−1.589	12	0.138	
*GST T1*	0.4367	0.7766	−1.993	7	0.086	
*GST T7*	0.0024	0.0108	−0.447	-	0.701	
*EST F*	0.0066	0.0162	−1.853	-	0.073	
***EST 1***	**2.76 × 10^−4^**	**7.13 × 10^−5^**	**5.654**	**10**	0.0001 *	***48 h***
*SUR*	0.0008	0.0011	−1.081	12	0.301	
***ABCG1***	**0.0028**	**0.0106**	**−7.336**	**12**	<0.0001 *	***120 h***
***ABCA3***	**0.0180**	**0.0328**	**−3.976**	**12**	0.001 *	***120 h***
***L259***	**0.0036**	**0.0137**	**−2.619**	**-**	0.009 *	***120 h***
*MDR49*	0.0063	0.0173	−1.853	-	0.073	
*CP6A9*	0.0009	0.0102	−1.597	-	0.110	
*CP313*	0.0113	0.0391	−2.265	6	0.061	
***CP134***	**0.0015**	**0.0308**	**−2.668**	**6**	0.036 *	***120 h***
*CP4D8*	0.0004	0.0078	−1.118	6	0.306	
***CP6G1***	**0.0017**	**0.1161**	**−3.003**	**-**	0.003 *	***120 h***
***C12E1***	**0.0161**	**0.0827**	**−2.491**	**6**	0.046 *	***120 h***
***CP6T1A***	**0.0003**	**0.0114**	**−2.108**	**-**	0.040 *	***120 h***
***CP6T1B***	**0.0005**	**0.0103**	**−2.236**	**-**	0.029 *	***120 h***
***C12B2***	**0.0207**	**0.0716**	**−3.879**	**7**	0.005 *	***120 h***
***C12B1***	**0.0010**	**0.0096**	**−2.747**	**-**	0.007 *	***120 h***
*CP306*	0.0019	0.0062	−0.971	6	0.368	
***CP304A***	**0.0003**	**0.0100**	**−3.002**	**-**	0.003 *	***120 h***
***C6A14***	**0.0083**	**0.0560**	**−3.903**	**6**	0.007 *	***120 h***
***CP4S3***	**0.0053**	**0.0381**	**−2.875**	**-**	0.004 *	***120 h***
*CP132*	0.0128	0.0249	−1.264	6	0.251	
*CP316*	0.0007	0.0012	−1.729	12	0.109	
***CP304B***	**0.0006**	**0.0172**	**−2.264**	**-**	0.018 *	***120 h***
*CP6G2*	0.0007	0.0084	−1.164	6	0.288	
**D-Larvae tissue** **Picked**	**48 h**	**120 h**				
*GST D1*	0.0835	0.1594	−1.981	-	0.053	
*GST T1*	0.4036	1.0905	−0.319	-	0.749	
*GST T7*	0.0017	0.0051	−1.214	-	0.225	
*EST F*	0.0071	0.0138	−1.550	6	0.168	
*EST 1*	0.0002	0.0001	1.766	7	0.117	
*SUR*	0.0006	0.0008	−1.331	6	0.227	
***ABCG1***	**0.0024**	**0.0095**	**−9.787**	**8**	<0.0001 *	***120 h***
*ABCA3*	0.0174	0.0269	−2.243	7	0.058	
*L259*	0.0037	0.0099	−1.089	-	0.277	
*MDR49*	0.0069	0.0173	−1.510	6	0.177	
*CP6A9*	0.0007	0.0059	−0.703	-	0.482	
***CP313***	**0.0091**	**0.0374**	**−2.970**	**6**	0.023 *	***120 h***
***CP134***	**0.0010**	**0.0270**	**−5.289**	**6**	0.001 *	***120 h***
*CP4D8*	0.0003	0.0038	−1.995	6	0.357	
***CP6G1***	**0.0009**	**0.0278**	**−3.130**	***-***	0.002 *	***120 h***
***C12E1***	**0.0153**	**0.0687**	**−2.630**	**6**	0.038 *	***120 h***
*CP6T1A*	0.0002	0.0048	−1.853	-	0.064	
*CP6T1B*	0.0003	0.0047	−1.981	-	0.055	
***C12B2***	**0.0194**	**0.0548**	**−3.612**	**6**	0.010 *	***120 h***
***C12B1***	**0.0009**	**0.0061**	**−2.492**	**-**	0.013 *	***120 h***
*CP306*	0.0018	0.0039	−0.708	6	0.504	
***CP304A***	**0.0003**	**0.0061**	**−2.489**	**-**	0.015 *	***120 h***
***C6A14***	**0.0075**	**0.0365**	**−2.942**	**6**	0.025 *	***120 h***
***CP4S3***	**0.0060**	**0.0216**	**−2.432**	**6**	0.048 *	***120 h***
*CP132*	0.0175	0.0213	−0.452	6	0.665	
*CP316*	0.0005	0.0008	−1.585	7	0.155	
***CP304B***	**0.0005**	**0.0101**	**−2.236**	**-**	0.029 *	***120 h***
*CP6G2*	0.0005	0.0046	−1.052	6	0.333	

## Data Availability

The data will be stored at the QUT data repository site, which will take a while to complete the whole procedure. The link of the data will be sent when available.

## Supplementary Materials

https://www.mdpi.com/article/10.3390/insects13050451/s1, Table S1: Three-way analysis of variance results for the analysis of *Bactrocera tryoni* larval survival in tomato fruit of two ripening stages (colour break and fully ripe), two cultivars (Roma and Cherry) and two picking states (picked or unpicked). Separate ANOVAs are presented for the fruit that were destructively sampled 48 and 120 h after larval inoculation.

## References

[1] McCall A.C., Fordyce J.A. (2010). Can optimal defence theory be used to predict the distribution of plant chemical defences?. J. Ecol..

[2] Rhoades D.F., Cates R.G., Wallace J.W., Mansell R.L. (1976). Toward a general theory of plant antiherbivore chemistry. Biochemical Interaction Between Plants and Insects.

[3] Van der Pijl L. (1982). Principles of Dispersal.

[4] Zangerl A.R., Rutledge C.E. (1996). The probability of attack and patterns of constitutive and induced defense: A test of optimal defense theory. Am. Nat..

[5] Cazetta E., Schaefer H.M., Galetti M. (2008). Does attraction to frugivores or defense against pathogens shape fruit pulp composition status?. Oecologia.

[6] Cipollini M.L., Levey D.J. (1997). Why are some fruits toxic? glycoalkaloids in solanum and fruit choice by vertebrates. Ecology.

[7] Salerno G., Rebora M., Piersanti A., Gorb E., Gorb S. (2020). Mechanical ecology of fruit-insect interaction in the adult Mediterranean fruit fly *Ceratitis capitata* (Diptera: Tephritidae). Zoology.

[8] Nevo O., Razafimandimby D., Jeffrey J.A.J., Schulz S., Ayasse M. (2018). Fruit scent as an evolved signal to primate seed dispersal. Sci. Adv..

[9] Nevo O., Razafimandimby D., Valenta K., Jeffrey J.A.J., Reisdorff C., Chapman C.A., Ganzhorn J.U., Ayasse M. (2019). Signal and reward in wild fleshy fruits: Does fruit scent predict nutrient content?. Ecol. Evol..

[10] Whitehead S.R., Quesada M.F.O., Bowers M.D. (2016). Chem tradeoffs in seed dispersal: Defensive metabolites in fruits deter consumption by mutualist bats. Oikos.

[11] Agrawal A.A., Gorski P.M., Tallamy D.W. (1999). Polymorphism in plant defense against herbivory: Constitutive and induced resistance in *Cucumis sativus*. J. Chem. Ecol..

[12] Farmer E.E. (2014). Leaf Defence.

[13] Paudel S., Lin P.-A., Foolad M.R., Ali J.G., Rajotte E.G., Felton G.W. (2019). Induced plant defenses against herbivory in cultivated and wild tomato. J. Chem. Ecol..

[14] War A.R., Taggar G.K., Hussain B., Taggar M.S., Nair R.M., Sharma H.C. (2018). Plant defence against herbivory and insect adaptations. AoB Plants.

[15] Diezel C., Allmann S., Baldwin I.T. (2011). Mechanisms of optimal defense patterns in *Nicotiana attenuata*: Flowering attenuates herbivory-elicited ethylene and jasmonate signalling. J. Integr. Plant Biol..

[16] Schiestl F.P., Kirk H., Bigler L., Cozzolino S., Desurmont G.A. (2014). Herbivory and floral signaling: Phenotypic plasticity and tradeoffs between reproduction and indirect defense. New Phytol..

[17] Gogi M.D., Ashfaq M., Arif M.J., Sarfraz R.M., Nawab N.N. (2010). Investigating phenotypic structures and allelochemical compounds of the fruits of *Momordica charantia* L genotypes as sources of resistance against *Bactrocera cucurbitae* (Coquillett) (Diptera: Tephritidae). Crop Prot..

[18] Staub C.G., De Lima F., Majer J.D. (2008). Determination of host status of citrus fruits against the Mediterranean fruit fly, *Ceratitis capitata* (Wiedemann) (Diptera: Tephritidae). Aust. J. Entomol..

[19] Cipollini M.L. (2000). Secondary metabolites of vertebrate-dispersed fruits: Evidence for adaptive functions. Rev. Chil. Hist. Nat..

[20] Herrera C.M. (1982). Defense of ripe fruit from pests: Its significance in relation to plant-disperser interactions. Am. Nat..

[21] Papanastasiou S.A., Bali E.-M.D., Ioannou C.S., Papachristos D.P., Zarpas K.D., Papadopoulos N.T. (2017). Toxic and hormetic-like effects of three components of citrus essential oils on adult Mediterranean fruit flies (*Ceratitis capitata*). PLoS ONE.

[22] Salvatore A., Borkosky A., Willink E., Bardón A. (2004). Toxic effects of lemon peel constituents on *Ceratitis capitata*. J. Chem. Ecol..

[23] Davis T.S., Garczynski S.F., Stevens-Rumann C., Landolt P.J. (2013). A test of fruit varieties on entry rate and development by neonate larvae of the codling moth, *Cydia pomonella*. Entomol. Exp. Appl..

[24] Greany P., Styer S., Davis P., Shaw P., Chambers D. (1983). Biochemical resistance of citrus to fruit flies Demonstration and elucidation of resistance to the Caribbean fruit fly, *Anastrepha suspensa*. Entomol. Exp. Appl..

[25] Muthuthantri S., Clarke A.R., Hayes R.A., Kevin J. (2015). Effect of citrus peel chemicals on *Bactrocera tryoni* larval survival. Acta Hortic..

[26] Papachristos D.P., Kimbaris A.C., Papadopoulos N.T., Polissiou M.G. (2009). Toxicity of citrus essential oils against *Ceratitis capitata* (Diptera: Tephritidae) larvae. Ann. Appl. Biol..

[27] Kreuger B., Potter D.A. (1994). Changes in saponins and tannins in ripening holly fruits and effects of fruit consumption on nonadopted insect herbivores. Am. Midl. Nat..

[28] Lasa R., Tadeo E., Dinorín L.A., Lima I., Williams T. (2017). Fruit firmness, superficial damage, and location modulate infestation by *Drosophila suzukii* and *Zaprionus indianus*: The case of guava in Veracruz, Mexico. Entomol. Exp. Appl..

[29] Mulatu B., Applebaum S., Kerem Z., Coll M. (2006). Tomato fruit size, maturity and α-tomatine content influence the performance of larvae of potato tuber moth *Phthorimaea operculella* (Lepidoptera: Gelechiidae). Bull. Entomol. Res..

[30] Rashmi M.A., Verghese A., Shivashankar S., Chakravarthy A.K., Sumathi M., Kandakoors S. (2017). Does change in tannin content in mango (*Mangifera indica*) fruits influence the extent of fruit fly (*Bactrocera dorsalis* Hendel) herbivory?. J. Entomol. Zool. Stud..

[31] Schaefer H.M., Schmidt V., Winkler H. (2003). Testing the defence trade-off hypothesis: How contents of nutrients and secondary compounds affect fruit removal. Oikos.

[32] Baba V.Y., Constantino L.V., Ivamoto S.T., Moreira A.F.P., Madeira T.B., Nixdorf S.L., Rodrigues R., Gonçalves L.S.A. (2019). *Capsicum-Colletotrichum* interaction: Identification of resistance sources and quantification of secondary metabolites in unripe and ripe fruits in response to anthracnose infection. Sci. Hortic..

[33] Cota I., Troncoso-Rojas R., Sotelo-Mundo R., Sánchez-Estrada A., Tiznado-Hernández M. (2007). Chitinase and β-1, 3-glucanase enzymatic activities in response to infection by *Alternaria alternata* evaluated in two stages of development in different tomato fruit varieties. Sci. Hortic..

[34] González G., Fuentes L., Moya-León M.A., Sandoval C., Herrera R. (2013). Characterization of two *PR* genes from *Fragaria chiloensis* in response to *Botrytis cinerea* infection: A comparison with *Fragaria* × *ananassa*. Physiol. Mol. Plant Pathol..

[35] Mikulic-Petkovsek M., Schmitzer V., Jakopic J., Cunja V., Veberic R., Munda A., Stampar F. (2013). Phenolic compounds as defence response of pepper fruits to *Colletotrichum coccodes*. Physiol. Mol. Plant Pathol..

[36] Mongkolporn O., Montri P., Supakaew T., Taylor P.W. (2010). Differential reactions on mature green and ripe chili fruit infected by three *Colletotrichum* spp. Plant Dis..

[37] Rao S., Nandineni M.R. (2017). Genome sequencing and comparative genomics reveal a repertoire of putative pathogenicity genes in chilli anthracnose fungus C*olletotrichum truncatum*. PLoS ONE.

[38] Salzman R.A., Tikhonova I., Bordelon B.P., Hasegawa P.M., Bressan R.A. (1998). Coordinate accumulation of antifungal proteins and hexoses constitutes a developmentally controlled defense response during fruit ripening in grape. Plant Physiol..

[39] Shah P., Powell A.L., Orlando R., Bergmann C., Gutierrez-Sanchez G. (2012). Proteomic analysis of ripening tomato fruit infected by *Botrytis cinerea*. J. Proteome Res..

[40] Van Loon L., Van Strien E. (1999). The families of pathogenesis-related proteins, their activities, and comparative analysis of PR-1 type proteins. Physiol. Mol. Plant Pathol..

[41] Vilanova L., Wisniewski M., Norelli J., Viñas I., Torres R., Usall J., Phillips J., Droby S., Teixidó N. (2014). Transcriptomic profiling of apple in response to inoculation with a pathogen (*Penicillium expansum*) and a non-pathogen (*Penicillium digitatum)*. Plant Mol. Biol. Rep..

[42] Wojciechowska E., Weinert C.H., Egert B., Trierweiler B., Schmidt-Heydt M., Horneburg B., Graeff-Hönninger S., Kulling S.K., Geisen R. (2014). Chlorogenic acid, a metabolite identified by untargeted metabolome analysis in resistant tomatoes, inhibits the colonization by *Alternaria alternata* by inhibiting alternariol biosynthesis. Eur. J. Plant Pathol..

[43] Anand T., Bhaskaran R., Raguchander T., Samiyappan R., Prakasam V., Gopalakrishnan C. (2009). Defence responses of chilli fruits to *Colletotrichum capsici* and *Alternaria alternata*. Biol. Plant..

[44] Ruelas C., Tiznado-Hernández M., Sánchez-Estrada A., Robles-Burgueno M., Troncoso-Rojas R. (2006). Changes in phenolic acid content during *Alternaria alternata* infection in tomato fruit. J. Phytopathol..

[45] Alkan N., Friedlander G., Ment D., Prusky D., Fluhr R. (2015). Simultaneous transcriptome analysis of *Colletotrichum gloeosporioides* and tomato fruit pathosystem reveals novel fungal pathogenicity and fruit defense strategies. New Phytol..

[46] Blanco-Ulate B., Vincenti E., Powell A., Cantu D. (2013). Tomato transcriptome and mutant analyses suggest a role for plant stress hormones in the interaction between fruit and *Botrytis cinerea*. Front. Plant Sci..

[47] Corrado G., Alagna F., Rocco M., Renzone G., Varricchio P., Coppola V., Coppola M., Garonna A., Baldoni L., Scaloni A. (2012). Molecular interactions between the olive and the fruit fly *Bactrocera oleae*. BMC Plant Biol..

[48] Christenson L., Foote R.H. (1960). Biology of fruit flies. Annu. Rev. Entomol..

[49] Clarke A.R. (2019). Biology and Management of Bactrocera and Related Fruit Flies.

[50] Clarke A.R., Armstrong K.F., Carmichael A.E., Milne J.R., Raghu S., Roderick G.K., Yeates D.K. (2005). Invasive phytophagous pests arising through a recent tropical evolutionary radiation: The *Bactrocera dorsalis* complex of fruit flies. Annu. Rev. Entomol..

[51] Jessup A., Dominiak B., Woods B., De Lima C., Tomkins A., Smallridge C., Vreysen M.J.B., Robinson A.S., Hendrichs J. (2007). Area-wide management of fruit flies in Australia. Area-Wide Control of Insect Pests.

[52] Mau R.F.L., Jang E.B., Vargas R.I., Vreysen M.J.B., Robinson A.S., Hendrichs J. (2007). The Hawaii area-wide fruit fly pest management programme: Influence of partnerships and a good education programme. Area-Wide Control of Insect Pests.

[53] Hawkes N.J., Janes R.W., Hemingway J., Vontas J. (2005). Detection of resistance-associated point mutations of organophosphate-insensitive acetylcholinesterase in the olive fruit fly, *Bactrocera oleae* (Gmelin). Pestic Biochem. Physiol..

[54] Jin T., Zeng L., Lin Y., Lu Y., Liang G. (2011). Insecticide resistance of the oriental fruit fly, *Bactrocera dorsalis* (Hendel) (Diptera: Tephritidae), in mainland China. Pest Manag. Sci..

[55] Vontas J., Hernández-Crespo P., Margaritopoulos J.T., Ortego F., Feng H.T., Mathiopoulos K.D., Hsu J.C. (2011). Insecticide resistance in tephritid flies. Pestic Biochem. Physiol..

[56] Dominiak B.C. (2019). Components of a systems approach for the management of Queensland fruit fly *Bactrocera tryoni* (Froggatt) in a post dimethoate fenthion era. J. Crop Prot..

[57] Aluja M., Arredondo J., Díaz-Fleischer F., Birke A., Rull J., Niogret J., Epsky N. (2014). Susceptibility of 15 mango (Sapindales: Anacardiaceae) cultivars to the attack by *Anastrepha ludens* and *Anastrepha obliqua* (Diptera: Tephritidae) and the role of underdeveloped fruit as pest reservoirs: Management implications. J. Econ. Entomol..

[58] Muthuthantri S., Clarke A.R. (2012). Five commercial citrus rate poorly as hosts of the polyphagous fruit fly *Bactrocera tryoni* (Froggatt) (Diptera: Tephritidae) in laboratory studies. Aust. J. Entomol..

[59] Papachristos D.P., Papadopoulos N.T., Nanos G.D. (2008). Survival and development of immature stages of the Mediterranean fruit fly (Diptera: Tephritidae) in citrus fruit. J. Econ. Entomol..

[60] Rattanapun W., Amornsak W., Clarke A.R. (2009). *Bactrocera dorsalis* preference for and performance on two mango varieties at three stages of ripeness. Entomol. Exp. Appl..

[61] Mateos M., Martinez Montoya H., Lanzavecchia S.B., Conte C., Guillén K., Morán-Aceves B.M., Toledo J., Liedo P., Asimakis E.D., Doudoumis V. (2020). *Wolbachia pipientis* associated with tephritid fruit fly pests: From basic research to applications. Front Microbiol.

[62] Armstrong J.W., Mitchell W.C., Farias G.J. (1983). Resistance of ‘Sharwil’ avocados at harvest maturity to infestation by three fruit fly species (Diptera: Tephritidae) in Hawaii. J. Econ. Entomol..

[63] Liquido N.J., Chan H.T., McQuate G.T. (1995). Hawaiian tephritid fruit flies (Diptera): Integrity of the infestation-free quarantine procedure for ‘Sharwil’avocado. J. Econ. Entomol..

[64] Papadopoulos N.T., Papachristos D.P., Ioannou C. (2015). Citrus fruits and the Mediterranean fruit fly. Acta Hortic..

[65] Nehra S., Singh S., Samota R.G., Choudhary S.K., Choudhary A.L. (2019). Screening of round gourd varieties for resistance against fruit fly, *Bactrocera cucurbitae* (Coquillett). J. Pharmacogn. Phytochem..

[66] Bower C.C. (1977). Inhibition of larval growth of the Queensland fruit fly, *Dacus tryoni* (Diptera: Tephritidae) in apples. Ann. Entomol. Soc. Am..

[67] Reissig W. (1979). Survival of apple maggot larvae, *Rhagoletis pomonella* (Diptera: Tephritidae), in picked and unpicked apples. Can. Entomol..

[68] Balagawi S., Vijaysegaran S., Drew R.A., Raghu S.J. (2005). Influence of fruit traits on oviposition preference and offspring performance of *Bactrocera tryoni* (Froggatt) (Diptera: Tephritidae) on three tomato (*Lycopersicon lycopersicum*) cultivars. Aust. J. Entomol..

[69] Roohigohar S., Prentis P.J., Clarke A.R. (2020). Effect of tomato fruit cultivar and ripening stage on *Bactrocera tryoni* (Froggatt) egg and larval survival. J. Appl. Entomol..

[70] Roohigohar S., Clarke A.R., Prentis P.J. (2021). Gene selection for studying frugivore-plant interactions: A review and an example using Queensland fruit fly in tomato. PeerJ.

[71] Heather N.W., Corcoran R.J., Singh P., Moore R.F. (1985). Dacus tryoni. Handbook of Insect Rearing.

[72] Sargent S.A. (1997). Tomato Production Guide for Florida: Harvest and Handling.

[73] Leach P., Clarke A.R. (2019). Phytosanitary measures. Biology and Management of Bactrocera and Related Fruit Flies.

[74] Sokal R.R., Rohlf F.J. (2012). Biometry: The Principles and Practice of Statistics in Biological Research.

[75] Graham M.H., Edwards M.S. (2001). Statistical significance versus fit: Estimating the importance of individual factors in ecological analysis of variance. Oikos.

[76] Pfaffl M.W., Dorak M.T. (2007). Relative quantification. Real-Time PCR.

[77] Ganger M.T., Dietz G.D., Ewing S.J. (2017). A common base method for analysis of qPCR data and the application of simple blocking in qPCR experiments. BMC Bioinform..

[78] Orcan F. (2020). Parametric or non-parametric: Skewness to test normality for mean comparison. Int. J. Assess. Tool Educ..

[79] Arnó J., Solà M., Riudavets J., Gabarra R. (2016). Population dynamics, non-crop hosts, and fruit susceptibility of *Drosophila suzukii* in Northeast Spain. J. Pest Sci..

[80] Ben-Yosef M., Pasternak Z., Jurkevitch E., Yuval B. (2015). Symbiotic bacteria enable olive fly larvae to overcome host defences. R. Soc. Open Sci..

[81] Díaz-Fleischer F., Aluja M. (2003). Clutch size in frugivorous insects as a function of host firmness: The case of the tephritid fly *Anastrepha ludens*. Ecol. Entomol..

[82] Joachim-Baravo I.S., Fernandes O.A., De Bortoli S.A., Zucoloto F.S. (2001). Oviposition behavior of *Ceratitis capitata* Wiedemann (Diptera: Tephritidae): Association between oviposition preference and larval performance in individual females. Neotrop. Entomol..

[83] Ishiguri Y., Toyoshima S. (2006). Larval survival and development of the peach fruit moth, *Carposina sasakii* (Lepidoptera: Carposinidae), in picked and unpicked apple fruits. Appl. Entomol. Zool..

[84] Alkan N., Fortes A.M. (2015). Insights into molecular and metabolic events associated with fruit response to post-harvest fungal pathogens. Front. Plant Sci..

[85] Beno-Moualem D., Prusky D. (2000). Early events during quiescent infection development by *Colletotrichum gloeosporioides* in unripe avocado fruits. Phytopathology.

[86] Birnbaum S.S., Rinker D.C., Gerardo N.M., Abbot P. (2017). Transcriptional profile and differential fitness in a specialist milkweed insect across host plants varying in toxicity. Mol. Ecol..

[87] Huang X., Lv S., Zhang Z., Chang B.H. (2020). Phenotypic and transcriptomic response of the grasshopper *Oedaleus asiaticus* (Orthoptera: Acrididae) to toxic rutin. Front. Physiol..

[88] Misra J.R., Horner M.A., Lam G., Thummel C.S. (2011). Transcriptional regulation of xenobiotic detoxification in *Drosophila*. Genes Dev..

[89] Scanlan J.L., Gledhill-Smith R.S., Battlay P., Robin C. (2020). Genomic and transcriptomic analyses in *Drosophila* suggest that the ecdysteroid kinase-like (EcKL) gene family encodes the ‘detoxification-by-phosphorylation’ enzymes of insects. Insect Biochem. Mol. Biol..

[90] Xu C., Li C.Y.T., Kong A.N.T. (2005). Induction of phase I, II and III drug metabolism/transport by xenobiotics. Arch. Pharm. Res..

[91] Després L., David J.P., Gallet C. (2007). The evolutionary ecology of insect resistance to plant chemicals. Trends Ecol. Evol..

[92] Hoang K., Matzkin L.M., Bono J.M. (2015). Transcriptional variation associated with cactus host plant adaptation in *Drosophila mettleri* populations. Mol. Ecol..

[93] Pavlidi N., Gioti A., Wybouw N., Dermauw W., Ben-Yousef M., Tuval B., Jurkevich E., Kampouraki A., Van Leeuwen T., Vontas J. (2017). Transcriptomic responses of the olive fruit fly *Bactrocera oleae* and its symbiont *Candidatus erwinia dacicola* to olive feeding. Sci. Rep..

[94] Tan W.H., Acevedo T., Harris E.V., Alcaide T.Y., Walters J.R., Hunter M.D., Gerardo N.M., de Roode J.C. (2019). Transcriptomics of monarch butterflies (*Danaus plexippus*) reveals that toxic host plants alter expression of detoxification genes and down-regulate a small number of immune genes. Mol. Ecol..

[95] Friedman M. (2002). Tomato glycoalkaloids: Role in the plant and in the diet. J. Agric. Food Chem..

[96] Chan H.T., Tam S.Y.T. (1985). Toxicity of α-tomatine to larvae of the Mediterranean fruit fly (Diptera: Tephritidae). J. Econ. Entomol..

[97] Baxter I. (2020). We aren’t good at picking candidate genes, and it’s slowing us down. Curr. Opin. Plant Biol..

[98] Kandoth P.K., Ranf S., Pancholi S.S., Jayanty S., Walla M.D., Miller W., Howe G.A., Lincoln D.E., Stratmann J.W. (2007). Tomato MAPKs *LeMPK1*, *LeMPK2*, and *LeMPK3* function in the systemin-mediated defense response against herbivorous insects. Proc. Natl. Acad. Sci. USA.

[99] Mahanil S., Attajarusit J., Stout M.J., Thipyapong P. (2008). Overexpression of tomato polyphenol oxidase increases resistance to common cutworm. Plant Sci..

[100] Xu S., Liao C.-J., Jaiswal N., Lee S., Yun D.-J., Lee S.Y., Garvey M., Kaplan I., Mengiste T. (2018). Tomato *PEPR1* ORTHOLOG RECEPTOR-LIKE KINASE1 regulates responses to systemin, necrotrophic fungi, and insect herbivory. Plant Cell.

[101] Zheng Y., Yang Y., Liu C., Chen L., Sheng J., Shen L. (2015). Inhibition of *SlMPK1*, *SlMPK2*, and *SlMPK3* disrupts defense signaling pathways and enhances tomato fruit susceptibility to *Botrytis cinerea*. J. Agric. Food Chem..

