# Supplementary material for: Fruit Fly Larval Survival in Picked and Unpicked Tomato Fruit of Differing Ripeness and Associated Gene Expression Patterns
Source: Insects. 2022 May 10;13(5):451. doi: 10.3390/insects13050451 (PMC9146954; doi:10.3390/insects13050451)
Supplement: Supplementary file 1 [file insects-13-00451-s001.zip › insects-1618569-supplementary.pdf]

Supplementary Table S1: Three-Way Analysis of variance results for the analysis of *Bactrocera tryoni* larval survival in tomato fruit of two ripening stages (colour-break and fully-ripe), two cultivars (Roma and Cherry) and two picking states (picked or unpicked). Separate ANOVAs are presented for fruit which was destructively sampled 48 and 120 hours after larval inoculation.

**Treatment/interaction**	**df**	**F**	**P**
**48 hours after inoculation**			
Ripening stage	**1, 72**	**7.73**	**<0.01**
Cultivar	1, 72	1.82	0.182
Picking status	1, 72	1.82	0.182
Ripening * Cultivar	1, 72	2.19	0.142
Ripening * Picking status	1, 72	2.50	0.117
Cultivar * Picking status	1, 72	0.77	0.380
Ripening * Cultivar * Picking s.	1, 72	0.08	0.778
**120 hours after inoculation**			
Ripening stage	1, 72	0.12	0.915
Cultivar	1, 72	0.56	0.453
Picking status	**1, 72**	**10.21**	**<0.01**
Ripening * Cultivar	1, 72	0.007	0.933
Ripening * Picking status	**1, 72**	**3.99**	**0.049**
Cultivar * Picking status	1, 72	0.26	0.608
Ripening * Cultivar * Picking s.	1, 72	0.47	0.495
